# *Engrailed2* modulates cerebellar granule neuron precursor proliferation, differentiation and insulin-like growth factor 1 signaling during postnatal development

**DOI:** 10.1186/2040-2392-5-9

**Published:** 2014-02-07

**Authors:** Ian T Rossman, Lulu Lin, Katherine M Morgan, Marissa DiGiovine, Elise K Van Buskirk, Silky Kamdar, James H Millonig, Emanuel DiCicco-Bloom

**Affiliations:** 1 Department of Neuroscience & Cell Biology, Robert Wood Johnson Medical School, Rutgers, The State University of New Jersey, 675 Hoes, Lane, Piscataway, NJ 08854, USA; 2 Department of Pediatrics (Child Neurology & Neurodevelopmental Disabilities), Robert Wood Johnson Medical School, Rutgers, The State University of New Jersey, New Brunswick, NJ 08901, USA; 3 Center for Pediatric Neurology & Neurosurgery, Cleveland Clinic, Cleveland, OH 44195, USA; 4 Department of Medicine, The Cancer Institute of New Jersey, Robert Wood Johnson Medical School, Rutgers, The State University of New Jersey, New Brunswick, NJ 08903, USA; 5 Department of Neurology, Children's Hospital of Philadelphia, Philadelphia, PA 19104, USA; 6 Department of Biology, Duke University, Durham, NC 27708, USA; 7 Center for Advanced Biotechnology and Medicine, Rutgers, The State University of New Jersey, Piscataway, NJ 08854, USA

**Keywords:** Autism, *Engrailed2*, IGF1, Cerebellum, Neurodevelopment, Cell cycle, Proliferation, Phospho-S6 kinase

## Abstract

**Background:**

The homeobox transcription factor *Engrailed2* (*En2*) has been studied extensively in neurodevelopment, particularly in the midbrain/hindbrain region and cerebellum, where it exhibits dynamic patterns of expression and regulates cell patterning and morphogenesis. Because of its roles in regulating cerebellar development and evidence of cerebellar pathology in autism spectrum disorder (ASD), we previously examined an *ENGRAILED2* association and found evidence to support *EN2* as a susceptibility gene, a finding replicated by several other investigators. However, its functions at the cell biological level remain undefined. In the mouse, *En2* gene is expressed in granule neuron precursors (GNPs) just as they exit the cell cycle and begin to differentiate, raising the possibility that *En2* may modulate these developmental processes.

**Methods:**

To define *En2* functions, we examined proliferation, differentiation and signaling pathway activation in *En2* knockout (KO) and wild-type (WT) GNPs in response to a variety of extracellular growth factors and following *En2* cDNA overexpression in cell culture. *In vivo* analyses of cerebellar GNP proliferation as well as responses to insulin-like growth factor-1 (IGF1) treatment were also conducted.

**Results:**

Proliferation markers were increased in KO GNPs *in vivo* and in 24-h cultures, suggesting *En2* normally serves to promote cell cycle exit. Significantly, IGF1 stimulated greater DNA synthesis in KO than WT cells in culture, a finding associated with markedly increased phospho-S6 kinase activation. Similarly, there was three-fold greater DNA synthesis in the KO cerebellum in response to IGF1 *in vivo*. On the other hand, KO GNPs exhibited reduced neurite outgrowth and differentiation. Conversely, *En2* overexpression increased cell cycle exit and promoted neuronal differentiation.

**Conclusions:**

In aggregate, our observations suggest that the ASD-associated gene *En2* promotes GNP cell cycle exit and differentiation, and modulates IGF1 activity during postnatal cerebellar development. Thus, genetic/epigenetic alterations of *EN2* expression may impact proliferation, differentiation and IGF1 signaling as possible mechanisms that may contribute to ASD pathogenesis.

## Background

The homeobox transcription factor *Engrailed2* (*En2*) has been studied extensively in neurodevelopment, particularly in the organization of the midbrain/hindbrain region and the cerebellum, where it exhibits dynamic, prenatal and postnatal expression patterns and complex regional functions [1,2]. Because of its roles in regulating cerebellar morphogenesis and Purkinje neuron development, and evidence of cerebellar pathology in human disease [3-6], we previously examined *ENGRAILED2’s* association with human autism spectrum disorder (ASD) and found evidence to support *EN2* as an ASD susceptibility gene. These results, initially observed in 167 families, were subsequently replicated in two additional data sets (518 families; P = 0.00000035), and six other groups have demonstrated *EN2* association with ASD [7-12].

In the developing mouse brain, *En2* restricts the fate of progenitor cells to a midbrain/hindbrain lineage and regulates cerebellar growth, patterning and connectivity. For example, *En2* deletion mutants exhibit hypoplastic cerebella with reduced numbers of Purkinje neurons as well as foliation defects and mistargeted spinocerebellar afferents [13-17]. Interestingly, transgenic misexpression of *En2* that increases gene expression in postnatal cerebellum also produced similar phenotypes, suggesting that proper levels of *En2* expression are required for normal development [18-20]. The fetal expression of *En2* in the mouse follows a complex pattern, initially expressed diffusely at the mid-hindbrain junction of the brainstem, but becoming increasingly restricted to the postnatal, developing cerebellum [1,21,22]. While the major cerebellar output neurons, the Purkinje cells, are generated prenatally, cerebellar expansion and its adult morphology result from massive proliferation of the granule neuron precursors (GNP) located in the postnatal external germinal layer (EGL) covering the cerebellum [13,16,18,23]. In the EGL, GNPs proliferate in the outer portion, whereas postmitotic precursors start differentiating in the inner layer. Significantly, *En2* gene expression is increased in GNP as they exit the cell cycle and begin to differentiate, raising the possibility that *En2* may participate in these developmental processes [14,15,24]. Because *En2* mutants have decreased numbers of Purkinje neurons that provide mitogenic growth factors for GNP proliferation, cerebellar hypoplasia has been considered a consequence of their deficiency. However, as an alternative hypothesis, *En2* expression in GNPs themselves may play a cell-autonomous role in regulating proliferation and differentiation. We now explore the function of *En2* during GNP development by comparing wild-type (WT) and knockout (KO) GNPs both in vivo and in culture, as well as by using *En2* overexpression constructs.

ASD is a highly heritable genetic disorder [25,26], with interactions between multiple susceptibility genes as well as environmental factors manifesting as diverse clinical presentations [27]. How individual susceptibility genes such as *EN2* contribute to disease risk (individually and in aggregate with other genes) remains to be elucidated. Cerebellar granule neurons are the largest population of neurons in the brain, and the only major population to continue neurogenesis postnatally, compared to more limited adult neurogenesis in the forebrain [23,28,29]. In humans, this process extends through infancy, the period when ASD symptoms first manifest. Significantly, multiple lines of evidence suggest that cerebellar dysfunction contributes to ASD symptomology [6]. Neuropathological studies demonstrate Purkinje neuron deficits in the majority of brains examined to date, whereas structural MRIs indicate that subsets of individuals have hypoplastic cerebellar vermis and others have enlarged cerebellar hemispheres [5,30,31]. Functional brain imaging studies suggest that cerebellar dysfunction contributes to the motor, cognitive and language deficits observed in ASD [4,32-35]. Thus, a disruption of early postnatal cerebellar development could potentially contribute to ASD pathogenesis. From this perspective, by defining the role of *En2* in postnatal GNP neurogenesis, we may identify cellular pathways by which variations in the levels of *En2* expression may contribute to disordered cerebellar development, potentially providing insight into ASD pathophysiology. We now define *En2* function in postnatal cerebellar development, specifically the period when GNPs transition from proliferation to differentiation concomitantly with their expression of *En2*[15,36]. We demonstrate that without *En2*, GNPs favor proliferation over differentiation, whereas *En2* overexpression promotes GNP cell cycle exit and differentiation. Furthermore, we identify previously unrecognized interactions between *En2* and IGF1 signaling.

## Methods

### Animals and genotyping

Time-mated Sprague-Dawley rats were obtained from Hilltop Lab Animals, Inc. (Scottdale, PA) and maintained on a 12:12 light:dark cycle with *ad libitum* Purina rat chow and water. Rats were killed by CO_2_ gas asphyxiation as approved by RWJMS IACUC. Male and female breeding pairs of C57BL6 J/129S2SV PAS mice heterozygous (HT) for a functional deletion of *En2*[13] were obtained from The Jackson Laboratory (no. 002657; Bar Harbor, ME) and maintained. An *En2* mutant colony was propagated by HT × HT intercrosses, and genotyping of the progeny was performed as described (jaxmice.jax.org). The mice were initially maintained as heterozygous matings, but, to generate adequate numbers of pups of known genotype for GNP cultures, WT × WT and KO × KO mating pairs were established.

All animal procedures were assessed and approved by the Robert Wood Johnson Medical School Institutional Animal Care and Use Committee. Animals were managed by Robert Wood Johnson Animal Facility, and maintenance, husbandry, transportation, housing and use were in compliance with the Laboratory Animal Welfare Act (PL 89–544; PL-91-579) and National Institutes of Health guidelines (Manual Chapter 4206).

### Recombinant DNA

A full-length *En2* cDNA was cloned from C57BL6 J/129S2SV PAS adult mouse cerebellum as described previously [7]. To identify transfected cells, the *En2* coding sequence was moved to the pCMS-EGFP expression vector (Clontech, Mountain View, CA) by EcoRI digestion and ligation. Orientation of positive clones was determined by Kpn1 digestion.

### Mouse and rat cerebellar granule neuron precursor (GNP) culture

Postnatal day (P)7 rat and WT or KO mice were decapitated and cerebellar GNPs isolated as described previously [37,38]. Briefly, 3–6 cleaned cerebella were incubated 3 min in trypsin-DNase solution (1% trypsin, 0.1% DNase, Worthington, Lakewood, NJ) and dissociated in DNase solution (0.05% in DMEM) by trituration. After pelleting, cells were filtered (30-*u*m nylon mesh; Tekton, Tarrytown, NY), resuspended and centrifuged at 3,200 rpm on a Percoll (Sigma, St. Louis, MO) 35:60% step gradient [39,40]. Cells at the 35:60% interface were collected and washed in phosphate buffer, then plated onto a poly-D-lysine (0.1 mg/ml)-coated 60-mm culture dish in defined medium (DM) composed of a 1:1 mixture of F12 and DMEM, 10 ng/ml insulin, 100 *u*g/ml transferrin, 10 mg/ml bovine serum albumin, 100 *u*M putrescine, 20 nM progesterone, 30 nM selenium, 6 mg/ml glucose, 50 U/ml penicillin and 50 *u*g/ml streptomycin. Unless stated otherwise, components were obtained from Sigma. After 1 h of preplating to remove adherent flat cells (<2% of cells), small round (granule) cells were dislodged by gentle pipetting and plated at ~10^4^-5 × 10^4^/cm^2^ on poly-D-lysine-coated 35-mm Nunc (Thermo Fischer Scientific, Rochester, NY) culture dishes or 24 multiwells. Cultures were maintained in a humidified 5% CO_2_/air incubator at 37°C.

For thymidine incorporation studies, cells were cultured in DM alone or with additional factors: high-dose insulin (10 *u*g/ml), 1–100 ng/ml IGF1 (Cell Sciences, Canton, MA), 0.1-3 *u*g/ml Shh-N (N-terminal fragment, R&D Systems, Minneapolis, MN), 10^-12^-10^-7^ M PACAP1-38 in 0.01 N acetic acid vehicle (American Peptide, Sunnyvale, CA), 10 ng/ml FGF2 (gift of Scios, Mountain View, CA), 10–30 ng/ml BDNF (PeproTech, Rocky Hill, NJ), 100 ng/ml EGF, 100–300 ng/ml VEGF or 10-30% conditioned media (CM) harvested from control- or Wnt3a-transfected fibroblasts.

For differentiation, cells were assessed live 24 h post plating by phase microscopy or were fixed in 4% cold paraformaldehyde and processed for immunocytochemistry, then assessed by phase or fluorescence microscopy. To assess neurites, cells bearing processes greater than two-cell soma diameters were assessed at 24 h in the live state, as reported previously [41], or following fixation and immunostaining against beta-III-tubulin as described below. Cells in two or three 1-cm rows (1.0-1.5% of the culture dish area) bearing neurites >2 cell soma were counted blind in two to four dishes per group, and experiments were performed three or more times.

### Assessment of DNA synthesis

Incorporation of ^3^H-deoxythymidine (^3^H-dT, 1*u*Ci/ml) was used to assess DNA synthesis, as described previously [38,42,43]. Cells were incubated with ^3^H-dT during the final 4 h of culture. DNA that had incorporated ^3^H-dT was collected onto glass fiber filters by a semiautomatic cell harvester (Skatron), and the radioligand was assessed by scintillation spectroscopy. To visualize cells synthesizing DNA, GNPs were exposed to the S-phase marker bromodeoxyuridine (BrdU) (10 *u*M, Sigma) during the final 4 h of incubation. After fixation, cells were either exposed to 2 N HCl (30 min) or pretreated with PBS/0.3% Triton X-100 and then DNaseI (500 U/ml, Worthington) [44] and subsequently processed for BrdU immunocytochemistry using monoclonal anti-BrdU (1:200; Dako, Carpinteria, CA) and visualized using a Vectastain avidin-biotin complex kit and Vector SG peroxidase substrate with a 3,3′-diaminobenzidine (DAB) chromogen (Vector Laboratories, Burlingame, CA) as previously described [45]. The labeling index, defined as the proportion of total cells incorporating BrdU into the nucleus, was determined by scoring the cells in five randomly selected, non-overlapping fields in each of the two to four dishes per group per experiment.

### cDNA transfection of GNPs

Rat and mouse GNPs were plated 2-4 × 10^5^cells/cm^2^ onto 12- or 25-mm glass coverslips (VWR International, West Chester, PA) pretreated with 2 N HCl for 30 min, rinsed in dH_2_O for 30 min, serially washed in 90% and 100% ethanol and fire-polished, then coated with poly-D-lysine (0.1 mg/ml) and fibronectin (1 *u*g/cm^2^, Sigma). Transfection media consisted of Neurobasal supplemented with 2% B27 (Invitrogen) containing glutamine (Gln, 2 mM), penicillin (50 U/ml), streptomycin (50 mg/ml), BSA (1 mg/ml), and either FGF2 (10 ng/ml) for rat GNPs or Shh (3 *u*g/ml) and BDNF (30 ng/ml) for mouse GNPs. For both species, 1 h after plating, cells were gently washed with Neurobasal supplemented with 10 *u*g/ml Gln, then incubated with transfection reaction media containing Neurobasal supplemented with 2% B27 and 10 *u*g/ml Gln, and either Lipofectamine Plus or Lipofectamine LTX Plus (Invitrogen) and 1 *u*g/ml pCMS-EGFP (GFP) or 1.2 *u*g/ml pCMS-EGFP-*En2* (En2). After 5 h, transfection reaction media were replaced with species-specific transfection media (as above) and cells were incubated an additional 24 h, then fixed in cold 4% paraformaldehyde. Successful transfection was determined by GFP autofluorescence, assessed by fluorescence microscopy prior to fixation or other procedures.

### Immunocytochemistry

Following fixation, cells were incubated 1 h at room temperature (RT) in 33% goat, rabbit or horse serum, then 1 h at RT or overnight at 4°C in one of the following primary antibodies diluted in PBS/0.3% Triton X-100, 2% serum and 0.05% NaN_3_: TuJ1, monoclonal mouse anti-beta-III-tubulin (1:9,000, Covance Research, Inc., Berkeley, CA), monoclonal mouse anti-PCNA (F2) (1:1,000, Santa Cruz, Inc., Santa Cruz, CA) or polyclonal chicken anti-GFP (1:5,000, Chemicon). Cells were then incubated 1 h with biotinylated horse anti-mouse secondary antibody and visualized with DAB chromogen or, alternatively, Alexafluor 488 (green) or 594 (red) fluorescent goat anti-mouse, goat anti-rabbit, rabbit anti-chicken, rabbit anti-mouse or rabbit anti-goat secondary antibody (1:1,000, Chemicon), followed by 5 min incubation with 4′,6-diamidino-2-phenylindole dihydrochloride (DAPI, 1 *u*g/ml, Sigma). Glass coverslips were inverted and mounted onto glass slides with Fluoromount-G (EMS, Hatfield, PA) and assessed by fluorescence microscopy. Cells were counted blind from two randomly selected fields within each of ten predetermined regions covering greater than 80% of the coverslip, from 2–4 coverslips per group per experiment. Images were acquired at 400× with and without background subtraction via an Apotome filter using Axiovision 4.5 software (Carl Zeiss Microimaging, Inc., Thornwood, NY).

### Protein collection and western blot analysis

Isolated P7 KO and WT GNPs (2.5 × 10^6^ cells/ml) were incubated in 4 ml DM on poly-D-lysine-coated (0.01 mg/ml) 60-mm dishes. After 4 h incubation, 10 ng/ml IGF1 was administered, followed by 15 min to 8 h incubation, then cells were washed with PBS, lysed by 70 *u*l lysis buffer containing 20 mM HEPES-KOH, pH 7.5, 5 mM KCl, 0.5 mM MgCl_2_ with 0.5 mM dithiothretiol (DTT), 2 mM phenylmethylsulfonylfluoride (PMSF), 1 mM leupeptin and 3.5 mg/ml aprotinin, and collected by scraping with rubber policeman. Samples were stored at -80°C. For blotting, defrosted samples were lysed by sonication and centrifuged, and the supernatant was normalized to 0.1 M NaCl. Protein concentrations were determined by Bio-Rad protein assay (Bio Rad Laboratories, Inc., Hercules, CA), and bovine serum albumin (BSA) dilutions served as standard curve. Protein extracts (50 *u*g per lane) were analyzed by 12% SDS-PAGE and electrically transferred to a polyvinylidenediflouride (PVDF) membrane. The membrane was blocked with 5% milk or 5% BSA and incubated with primary antibody against phospho- or total Akt, phospho- or total ERK1/2, phospho- or total GSK3beta, phospho- or total S6 kinase (1:1,000, Cell Signaling Technology, Danvers, MA) or total actin (1:2500, Chemicon), followed by monoclonal anti-mouse or polyclonal anti-rabbit horseradish peroxidase-conjugated secondary antibody, and visualized with an enhanced chemiluminescence system (Pierce, Rockford, IL), as previously reported [44,46].

### *In vivo* IGF1 administration and cerebellar ^3^H-dT incorporation

Groups of 3–5 P7 KO and WT pups were injected with PBS and 0.01 N HCl vehicle or IGF1 (10 *u*g/g, Cell Sciences) subcutaneously (sc) at time zero, followed at 6 h by ^3^H-dT (5 *u*Ci/g, sc), and were killed at 8 h. Cerebella were cleaned of meninges, removed from the brainstem, weighed in pre-tared microcentrifuge tubes and homogenized in dH_2_O (volume = mass × 5). An aliquot was removed for determination of total isotope uptake into the tissue. In an equal aliquot, DNA was precipitated with 10% trichloroacetic acid, sedimented by centrifugation, and washed by resuspension and resedimentation. The final pellet was dissolved and counted along with the original aliquot in a scintillation spectrophotometer. ^3^H-dT incorporation was defined as the fraction of total radionuclide taken up by the whole cerebellum that was present in the precipitated DNA and was reported as percent incorporation [47,48].

### *In vivo* BrdU immunohistochemistry

Two groups of three P7 KO and WT mice were injected sc with BrdU (50 *u*g/g) and killed at 2 h. Whole brains were removed and drop-fixed in fresh, cold 4% paraformaldehyde. Brains were serially dehydrated in 50, 60, 70, 80, 95 and 100% ethanol, 100% butanol and 100% xylene under vacuum and embedded in paraffin wax. Brains were then serially sectioned at 5 μm parasagittally, and sections were mounted on glass slides, with 5–6 sections/slide collected and numbered consecutively. Every fifth slide from WT and every fourth from KO (due to the smaller cerebellar size) plus two slides from either side of the central-most slide representing the central vermis were deparaffinized and rehydrated for anti-BrdU immunohistochemical processing. BrdU incorporated in DNA was detected by monoclonal antibody (Becton-Dickinson), amplified by biotinylated secondary antibody (Vectastain ABC kits), visualized by peroxidase reaction with DAB and subsequently counterstained with basic fuchsin. BrdU immunoreactive GNPs in the dorsal outer external germinal layer of lobules IV and VIII were counted by camera-lucida microscopy, and the BrdU labeling index quantified as the number of BrdU-positive cells/total cells × 100%. Four to eight sections from the hemispheres and four to eight sections from the vermis were counted for each pup [37,48].

### Statistical analyses

Data are expressed as mean ± SEM. Statistical comparisons were made by unpaired two-tailed Student’s *t* test or two-way ANOVA using Excel (Microsoft) or Vassarstats Website for Statistical Computation (http://www.vassarstats.net), respectively.

## Results

### Proliferation is enhanced *in vivo* in the absence of *En2*

**Figure 1:**
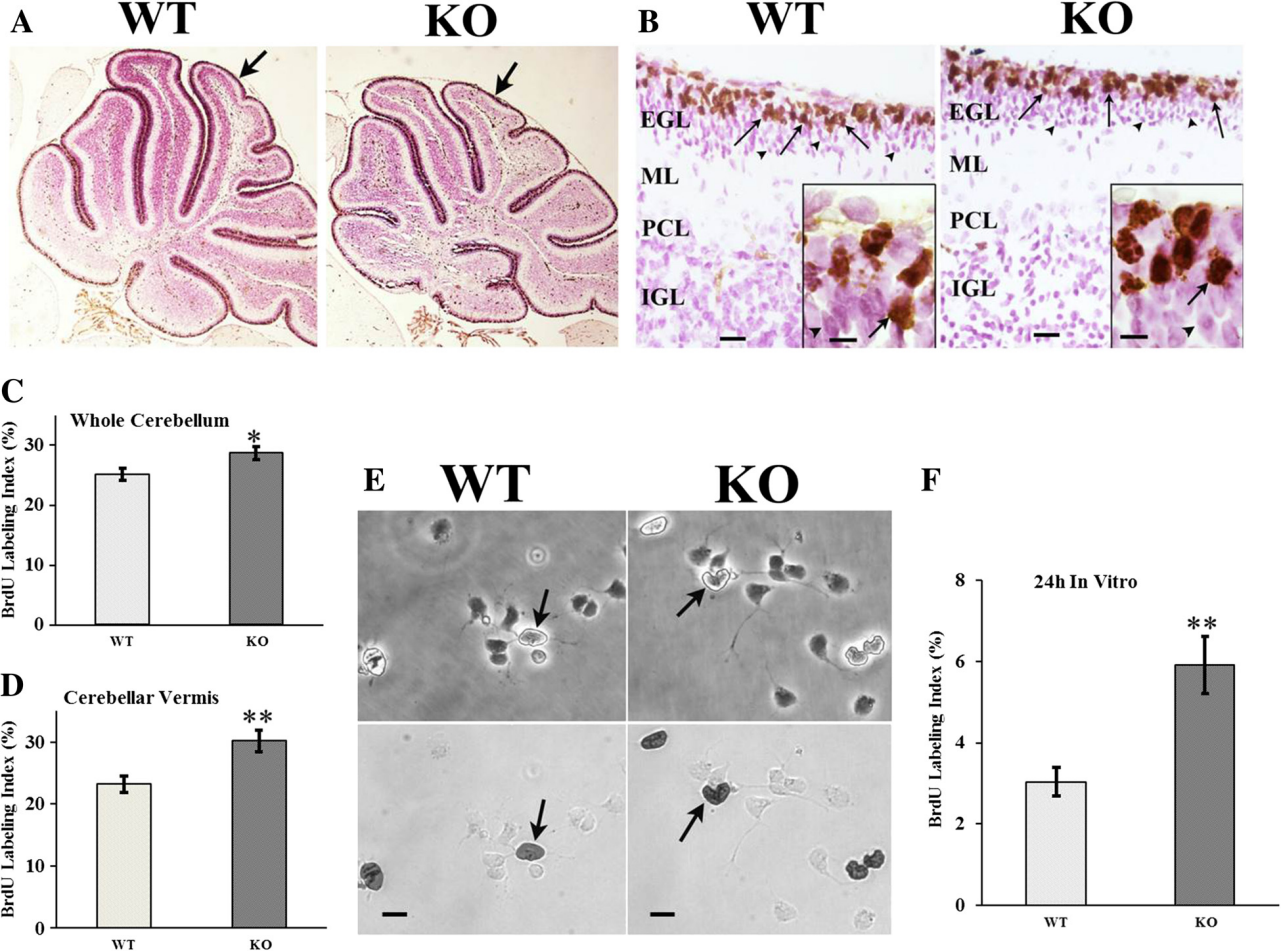
**GNP proliferation in the cerebellum *in vivo *and *in vitro*.** P7 WT and KO mice were injected with BrdU and killed 2 h later to perform BrdU LI analysis in the EGL. **(A)** Low magnification (4×) images of 5-μm sagittal sections from WT and KO cerebellar vermis are shown. As previously described [14,16], the KO cerebellum exhibits an overall smaller size. *Arrows* point to lobule IV. **(B)** At higher magnification, representative vermis sections show the middle top portion of the folium of lobule IV. Overall tissue cytoarchitecture appeared similar, and BrdU labeled GNPs were localized normally in both genotypes. *Arrows* denote proliferating GNPs in the outer EGL immunostained for S-phase marker BrdU; *arrowheads* denote post-mitotic GNPs in the inner EGL. *Insets* show cells in the EGL. *Bar* = 50 μm; *inset bar* = 10 μm. **(C)** When assessed across the entire extent of the cerebellum, the BrdU LI was moderately increased in the KO. **(D)** There was a greater increase in the KO LI when assessed within the cerebellar vermis. *N* = 4-5 animals per genotype; 8–14 sections were counted per entire cerebellum; 4–6 sections were counted per vermis region. **(E)** Twenty-four-hour cultures of WT and KO GNPs in control media are shown after fixation and BrdU immunocytochemisty. *Upper panels* show isolated GNPs under phase microscopy, whereas lower panels reveal brightfield images. *Arrows* point to cells in phase that exhibit BrdU immunostaining in *lower panels*. Bar = 10 μm. **(F)** The GNP BrdU LI *in vitro* was two-fold greater in the KO compared to WT cells. For each experiment, GNPs were isolated from groups of 4–6 mice per genotype. *N* = 3 culture dishes per genotype per experiment, and three experiments were performed. *EGL*: External germinal layer; *ML*: molecular Layer; *PCL*: Purkinje cell layer; *IGL*: inner granule layer. *t*-test: *p ≤ 0.05; **p ≤ 0.01.

Cerebellar GNPs increase expression of *En2* postnatally in the inner EGL, concurrent with their cell cycle exit and early differentiation; therefore, *En2* may be an important regulator of these processes. Using thymidine analog bromodeoxyuridine (BrdU) to label cells in S-phase, as previously published [48], we defined the labeling index [BrdU LI; (BrdU+/total cells) × 100%] of GNPs on postnatal day 7 (P7) of *En2* KO and WT cerebellum (Figure 1A,B). BrdU-labeled GNPs were localized normally in the outer EGL in both genotypes. The BrdU LI was increased by 15% in *En2* KO compared to the WT (Figure 1C), suggesting *En2* may regulate precursor proliferation. Furthermore, genotype-dependent differences were even greater when proliferation was assessed specifically in the centrally localized vermis, a region that may express higher *En2* levels [15,24]: the BrdU LI was 23% in the WT, whereas it was increased to 30% in the KO (Figure 1D). These results are consistent with a model in which *En2* normally suppresses S phase entry of GNP in the EGL.

### Differences in proliferation between *En2* KO and WT GNPs defined *in vivo* are preserved in culture

Differences in GNP proliferation *in vivo* may reflect the effects of distinct environmental signals such as growth factors derived from Purkinje neurons, which are reduced in the mutants, or alternatively, action of cell autonomous signals, such as *En2* expression [49]. Previously, we reported that in the absence of mitogens, specifically Shh, mouse GNPs in culture rapidly exit the cell cycle [38]. Therefore, WT and KO GNPs were isolated from the cerebellum and cultured at low density in defined media without growth factors, and DNA synthesis was assessed at 24 h by BrdU immunocytochemistry. The proportion of GNPs in mitotic S-phase was two-fold higher in *En2* KO cells (Figure 1E,F). Further, there were similar numbers of live KO and WT GNPs counted at 24 h (WT = 143 ± 10.4; KO = 120 ± 9.8, total cells/five fields ± SEM; *N* = 5-6 per genotype; *p* = 0.14). These *in vitro* results recapitulate the *in vivo* finding that *En2* KO GNPs fail to exit the cell cycle at the same rates as WT GNPs, an effect not likely due to changes in survival. These data also suggest that *En2* participates in cell-autonomous regulation of the GNP cell cycle, independent of extracellular growth factors in the EGL. However, this raises the question, does *En2* also function to modulate the effects of extracellular signals on cell cycle regulation?

### IGF1 stimulates enhanced proliferation in the absence of *En2* in culture and *in vivo*

Growth factors secreted by underlying Purkinje cells have been well characterized as regulators of GNP proliferation, differentiation and survival [49-52]; therefore, abnormal GNP responses to growth factor signaling in the absence of *En2* expression may contribute to the KO phenotype. To address this issue, we isolated *En2* KO and WT GNPs and compared proliferation, differentiation and survival in culture without and with developmentally relevant extracellular growth factors.

**Figure 2:**
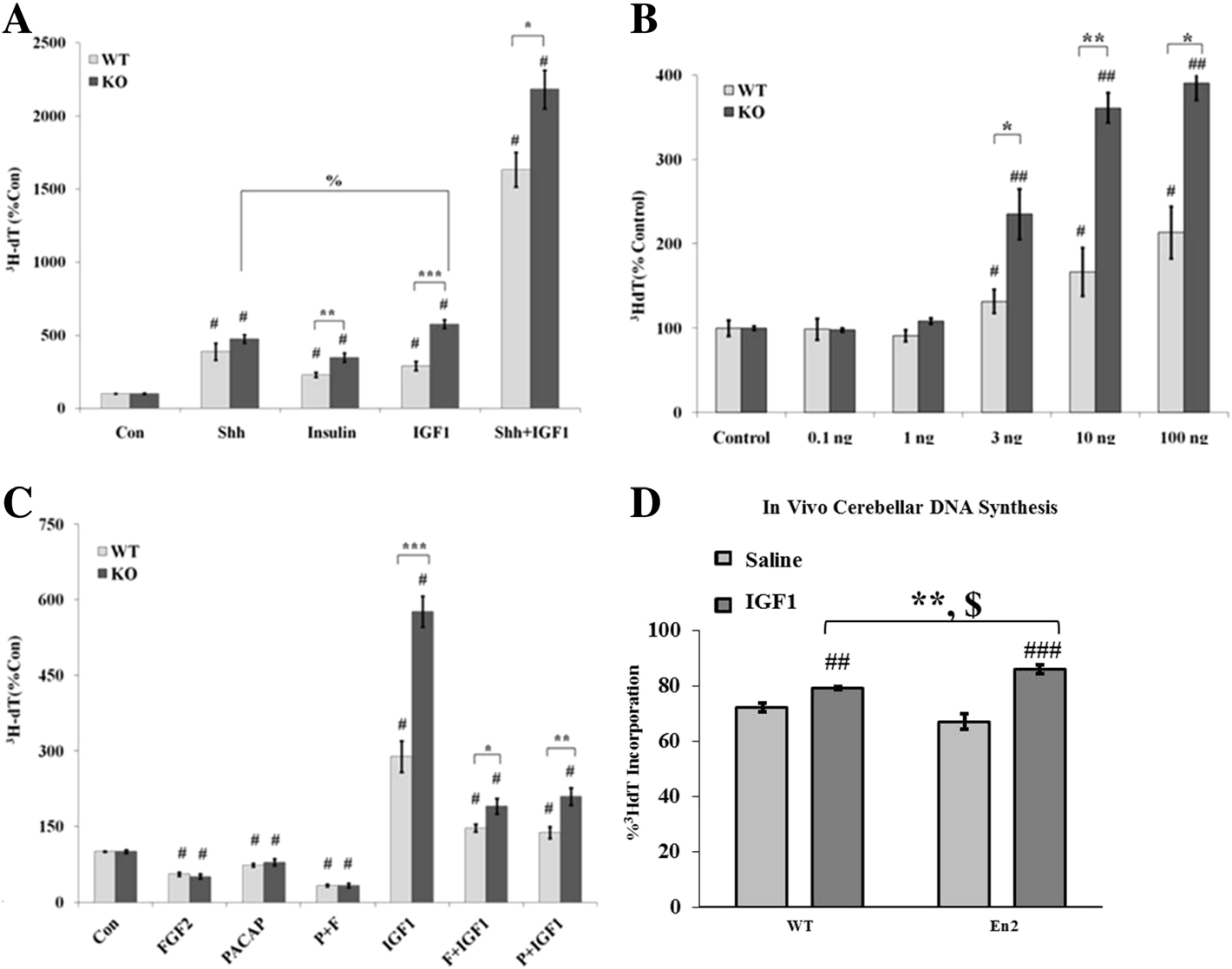
**IGF1 stimulates greater DNA synthesis in *En2 *KO GNPs in culture and *in vivo*. (A)** Mitogenic stimulation of KO and WT GNPs was measured in 24 h cultures using ^3^H-dT incorporation. IGF1 (10 ng/ml) and high-dose insulin (10 μg/ml) differentially increased KO DNA synthesis compared to WT, while Shh (3 μg/ml) elicited similar increases in both genotypes. Co-administration of IGF1 and Shh synergistically increased DNA synthesis, though differential genotype responses to IGF1 remained. Data presented as percent control ^3^H-dT incorporation ± SEM; control: 350–550 cpm/well; growth factors: 800–2,200 cpm/well; 4–6 mice/genotype/experiment; 3–6 wells/treatment/experiment, derived from three experiments. **(B)** IGF1 elicited differential genotype responses at doses above 1 ng/ml (Note: IGF1 elicited 3-4-fold increased KO DNA synthesis in Figure 2A-B; WT responses were less consistent across experiments.) *N* = 6-9 wells/group, from 3 experiments. **(C)** Anti-mitogens reduced DNA synthesis similarly across genotypes. FGF2 (10 ng/ml) and PACAP (10 nM) attenuated, but did not abolish, differential genotype responses to IGF1. *N* = 6-9 wells/group from three experiments. **(D)** In vivo, IGF1 (10μg/gbw) also differentially increased cerebellar ^3^H-dT incorporation (see Methods). ^3^H-dT incorporation after saline injection was not different between genotypes (WT = 73% + 1.6; KO = 66% + 3.5; *p* = 0.08). IGF1 significantly increased DNA synthesis over saline injection in both genotypes, however WT DNA synthesis increased 9.7% while KO DNA synthesis increased 28%. Two-way ANOVA yielded significant genotype × IGF1 interaction. KO: *N* = 9 saline, *N* = 6 IGF1; WT: *N* = 12 saline, *N* = 6 IGF1; *N* = 6 experiments, a minimum of one pup per genotype was injected with saline and IGF1 in each experiment. *Con*: Control; *F*: FGF2; *P*: PACAP: *t*-test *, compared between genotypes, *p* < 0.05; **p < 0.01; ***p < 0.001; *t*-test #, compared to genotype control, *p* < 0.05; ##*p* < 0.01; ###*p* < 0.001; %, compared within genotype, *p* < 0.05; $: two-way ANOVA, IGF1 F(1,29) = 28.5, *p* < 0.0001, genotype F(1,29) = 0.11, *p* = 0.74, genotype × IGF1 interaction F(1,29) = 4.88, *p* = 0.035.

Mitogenic growth factors, those that promote G1/S-phase transition, were added to defined media, and DNA synthesis in KO and WT GNPs was assessed by measuring ^3^H-deoxythymidine (^3^H-dT) incorporation. Sonic hedgehog (Shh), arguably the most well-recognized mitogen in postnatal cerebellar development [38,50,53-55], elicited identical increases in DNA synthesis in both genotypes (Figure 2A). However, insulin-like growth factor 1 (IGF1) and high-dose insulin, known to act through the IGFI receptor [56], elicited two-fold greater increases in DNA synthesis of KO GNPs (Figure 2A). This differential genotype response was apparent at physiologic concentrations of IGF1 and above (Figure 2B). Since the dose-response profiles to IGF1 were similar between genotypes, the differential mitogenic responses were not a function of the specific dose employed, and the receptor-ligand binding kinetics are apparently unchanged. Furthermore, Shh and IGF1 together increased DNA synthesis synergistically; however, the differential genotype-specific responses persisted (Figure 2A). This suggests IGF1 may specifically stimulate enhanced proliferation in the absence of *En2*. To further explore the specificity of IGF1 effects, anti-mitogenic stimuli, fibroblast growth factor-2 (FGF2) and pituitary adenylate cyclase activating peptide (PACAP), which block cell cycle progression in mouse GNPs [38], were added to defined media, and DNA synthesis was assessed (Figure 2C). FGF2 treatment reduced WT GNP incorporation by 50%, whereas PACAP elicited a 25% reduction, and both FGF2 and PACAP together reduced incorporation by 65%; virtually identical changes in DNA synthesis were observed in KO GNPs. Moreover, these anti-mitogenic signals attenuated IGF1-stimulated increases in DNA synthesis in both genotypes. Nonetheless, IGF1 stimulated greater DNA synthesis in KO GNPs even in the presence of these anti-mitogenic stimuli (Figure 2C). No other growth factors we tested elicited differential genotype-specific responses in DNA synthesis, including epidermal growth factor (EGF), brain-derived neurotrophic factor (BDNF) and vascular endothelial growth factor (VEGF), nor did Wnt-3a-conditioned media (data not shown). These data suggest IGF1 signaling may be differentially regulated in the absence of *En2* in cultured GNPs.

To determine whether IGF1 signaling is differentially regulated by the absence of *En2 in vivo*, we administered a single subcutaneous injection of IGF1 (10*u*g/gbw) to P7 KO and WT mice and measured total cerebellar tissue DNA synthesis using radiolabeled ^3^H-dT thymidine. As previously described for effects of peripherally injected bFGF on neonatal cerebellum [48], changes in the magnitude of ^3^H-dT thymidine incorporation into whole cerebellar homogenates (see [37], Figure one) closely paralleled changes in the labeling index of isolated GNPs (see [37], Figure four), supporting the value of this approach. With regard to IGF1, previous studies indicate that peripherally administered growth factor actively passes the blood-brain-barrier via a saturable transport mechanism and penetrates the brain parenchyma [41,57]. Preliminary studies conducted at 4, 8 and 12 h post-IGF1 injection, with a subcutaneous ^3^H-dT (5*u*Ci/g) injection 2 h prior to sacrifice, indicated the greatest increase in DNA synthesis was observed at 8 h. As we detected in culture, IGF1 significantly increased total cerebellar DNA synthesis in both genotypes *in vivo*. However, in the absence of *En2*, IGF1 elicited a far greater increase in cerebellar DNA synthesis, increasing ^3^H-dT incorporation by 28% over saline injection in the KO, whereas the increase was only 9.7% in the WT (Figure 2D). These data recapitulate *in vivo* that IGF1-induced mitogenesis is differentially regulated in the absence of *En2* and suggest *En2* negatively regulates IGF1 signaling during postnatal cerebellar development.

### Differentiation is diminished in the absence of *En2*

**Figure 3:**
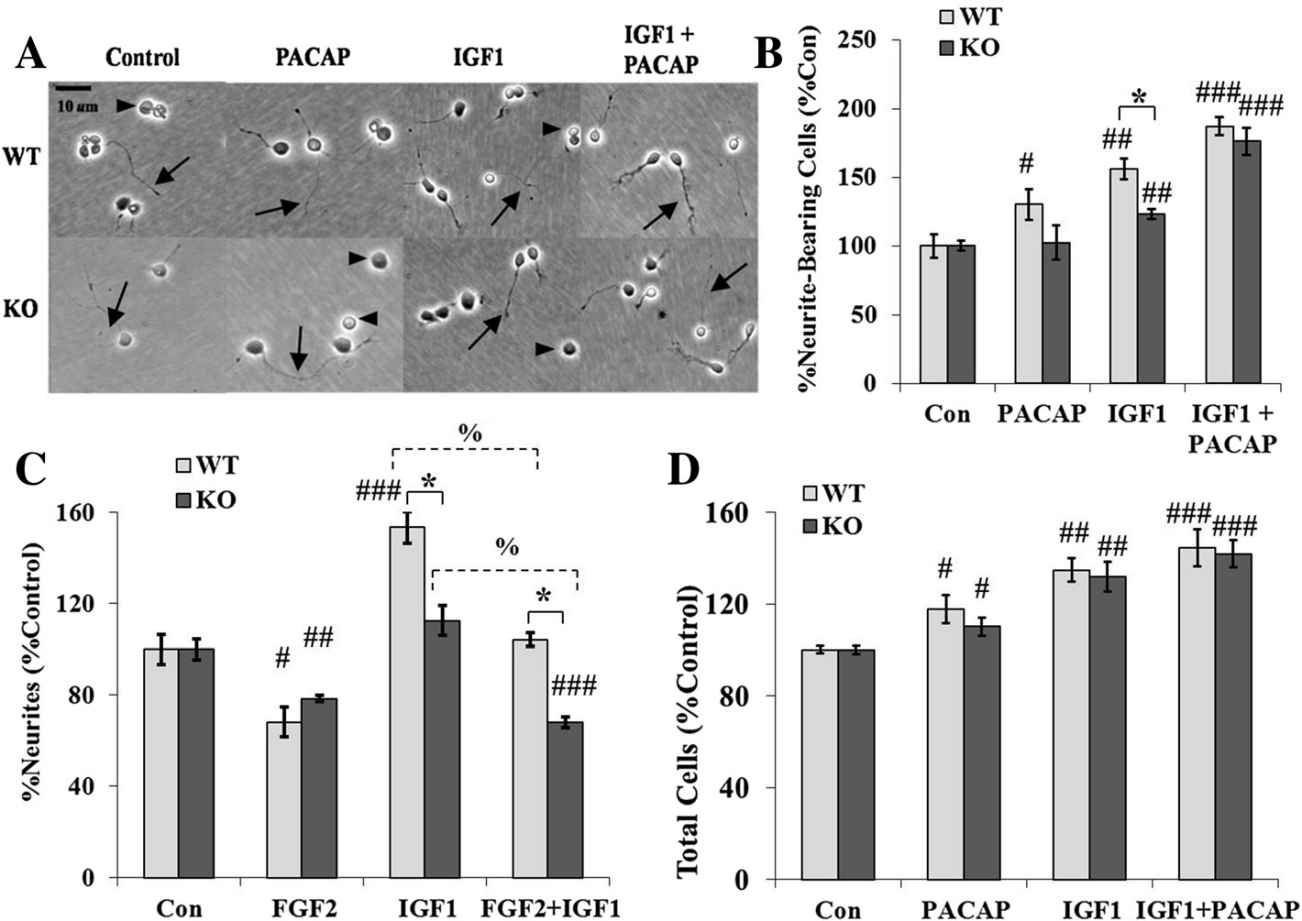
***En2 *KO GNPs exhibit normal survival but diminished response to neuritogenic signals compared to WT GNPs.** KO and WT GNPs were cultured in defined media without and with PACAP (10 nM), IGF1 (10 ng/ml) or both, and the percent of living, neurite-bearing cells was assessed under phase microscopy. **(A)** KO and WT GNP morphology was qualitatively similar in response to PACAP and IGF1, though numbers of neurite-bearing cells differed, as quantified in **B**-**C**. *Arrows* denote neurites extending many cell bodies from neuronal somas. *Arrowheads* identify cells without neurites >2 cell somas. **(B)** PACAP and IGF1 each induced fewer neurite-bearing KO GNPs compared to WT GNPs. However, in combination IGF1 and PACAP stimulated KO GNP neuritogenesis equivalent to WT. For each group, 2–3 dishes were assessed in each experiment for each genotype, derived from three experiments, yielding *N* = 6-9 dishes/group/genotype. **(C)** Anti-mitogenic growth factors failed to overcome differentiation deficits observed in the absence of *En2*. FGF2 reduced neurite outgrowth in both genotypes, whereas IGF1 again stimulated neuritogenesis in WT cells only. In combined factor treatment, WT cells exhibited outgrowth equivalent to control media, but KO cells displayed outgrowth inhibition similar to FGF2 treatment alone. These data suggest cell cycle exit and differentiation are mediated through different pathways in GNPs, and the absence of *En2* reduces IGF1 neuritogenic effects. *N* = 6-14 dishes/group/genotype. **(D)** Cell survival is similar in 24-h culture in WT and KO GNPs in response to trophic factors (compared to counts at 2 h; 200–400 cells counted/dish at 24 h), suggesting differences in DNA synthesis and neuritogenesis are unlikely to reflect differences in survival or death. *N* = 6-11 dishes/group/genotype. *t*-test *, compared between genotypes, *p* ≤ 0.05; *t*-test #, compared to genotype control, *p* ≤ 0.05; ## *p* ≤ 0.01; ### *p* ≤ 0.001; *t*-test %, compared within genotype, *p* ≤ 0.0001.

Postnatal *En2* expression begins in postmitotic GNPs in the inner EGL at, or just before, the start of differentiation and migration [15,24], suggesting this gene may regulate these processes. However, *En2* KO mice develop a mature, albeit smaller, IGL than the WT, suggesting *En2* may not be required for these processes but rather may modulate them. To address GNP differentiation in the absence of *En2*, KO and WT cells were cultured in defined media for 24 h without and with growth factors, and differentiation was assessed by quantifying neurite-bearing cells, as previously described [58] (Figure 3A). In media lacking growth factors, an identical percentage of WT and KO GNPs extended neurites (WT = 16.5% ± 1.88; KO = 16.8% ± 0.74; percent bearing neurites ± SEM; *p* = 0.86). When cultured with well-characterized neuritogenic factors PACAP or IGF1 [51,52], WT cells exhibited increased neurite outgrowth of 30% and 56%, respectively, over that occurring in defined media alone. However, *En2* KO GNPs failed to respond to PACAP, whereas IGF1 only increased KO neurite outgrowth by 23%, a 59% reduction (*p* ≤ 0.05) in IGF1-stimulated outgrowth compared to WT levels (Figure 3B). Thus, in the absence of *En2*, GNPs exhibit reduced growth factor-induced neurite outgrowth, suggesting that *En2* expression normally enhances differentiation. Interestingly, simultaneous administration of IGF1 and PACAP together “rescued” the KO GNP differentiation deficit, increasing neurite outgrowth to WT GNP levels (Figure 3B). These results may suggest there are convergent signaling pathways downstream of PACAP and IGF1 that together can promote GNP differentiation even in the absence of *En2*.

However, there may be an alternative model for the ability of PACAP plus IGF1 to overcome the neurite outgrowth deficiency observed in KO GNPs. Specifically, the anti-mitogenic effect of PACAP on both WT and KO GNPs, as above (Figure 2C), may allow cells to differentiate that would have otherwise continued to proliferate. Thus, any anti-mitogenic signal would be expected to promote IGF1-induced neuritogenesis. To address this issue, KO and WT GNPs were cultured with FGF2, another anti-mitogenic signal, and effects of IGF1 were examined. FGF2, a strong anti-mitogenic signal in both WT and KO GNPs (Figure 2C), elicited reductions in neurite outgrowth in both genotypes, suggesting it is not a pro-differentiation signal either (Figure 3C). FGF2 significantly attenuated IGF1-induced neurite outgrowth by more than 30% in both genotypes (Figure 3C). Thus, FGF2 represents both an anti-mitogenic and anti-neuritogenic signal that is equally active in both KO and WT GNPs. These findings are consistent with at least one report suggesting FGF2 inhibits neurite outgrowth in cerebellar GNPs [59]. Further, these data suggest KO GNP deficits in neurite outgrowth are not due to failed cell cycle exit, but rather a specific failure or delay in differentiation in the absence of *En2*.

In addition to regulating the cell cycle and differentiation, both PACAP and IGF1 exert trophic support for developing GNPs in the EGL [38,52]. Therefore, differences observed in DNA synthesis and neurite outgrowth between WT and *En2* KO GNPs could reflect differences in survival. To examine survival, cells were plated at low density (10,000 cells/cm^2^) to limit cell-cell contact and counted at 24 h in defined media without and with PACAP, IGF1, or PACAP and IGF1 together. Initial counts were taken at 2 h post plating to ensure equal numbers of cells were aliquoted and attached between genotypes. As reported previously, both PACAP and IGF1 increased the number of WT cells, and they exhibited a trend toward additivity when co-administered (Figure 3D). Significantly, *En2* KO cells responded identically to WT GNPs, exhibiting similar increases in cell numbers when incubated with PACAP, IGF1 and combined factors (Figure 3D). Thus, the absence of *En2* does not appear to be deleterious to GNP survival or to alter the trophic responses to growth factors. Further, these data suggest differences in DNA synthesis and neurite outgrowth observed between KO and WT GNPs are due to differentially regulated mechanisms underlying mitosis and differentiation, and not differences in cell survival or cell death.

### IGF1 signaling is altered in the absence of *En2*

**Figure 4:**
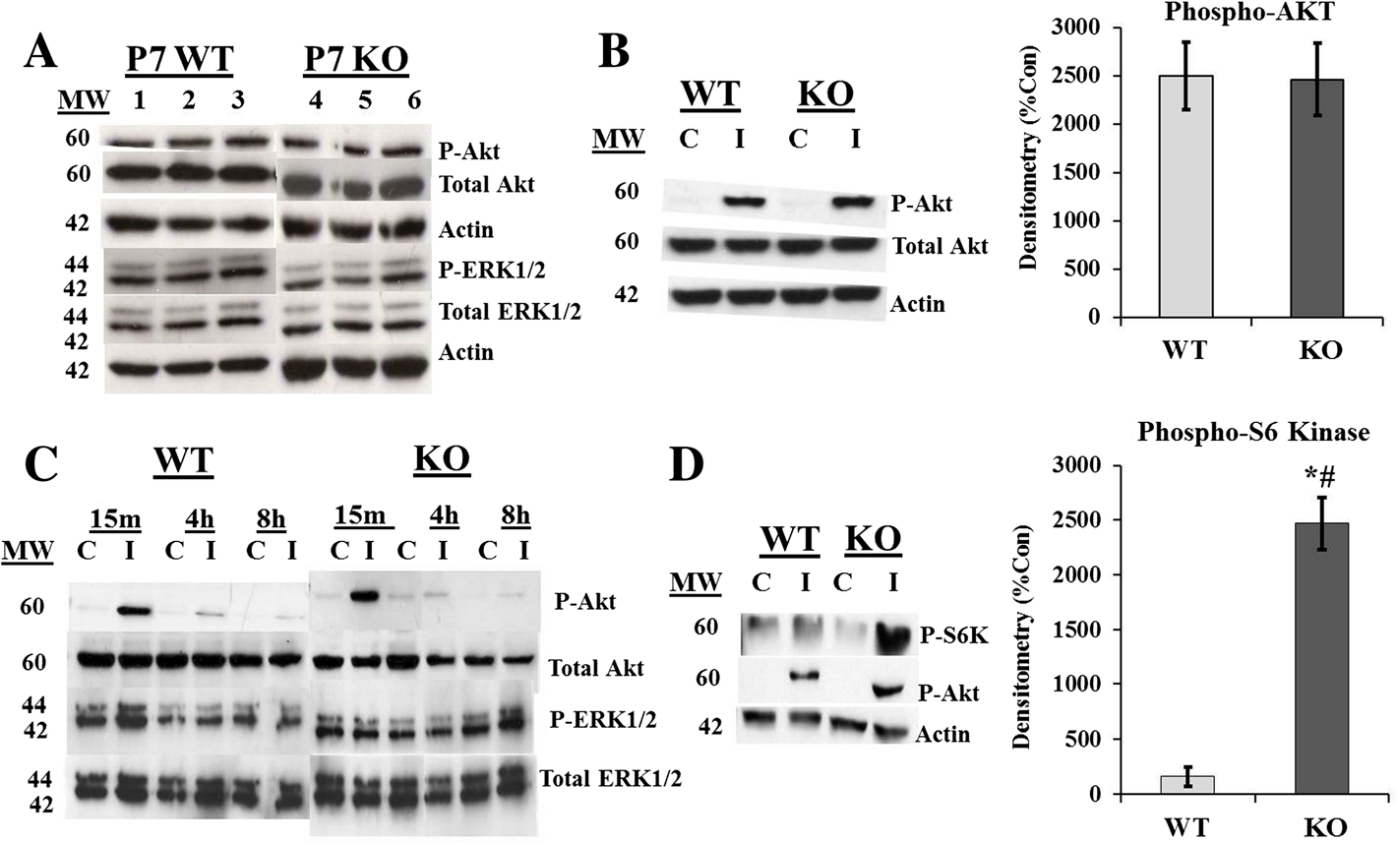
**IGF1 induces greater levels of phospho-S6 kinase in KO GNPs compared to WT cells. (A)** Untreated cerebellar lysates from freshly dissected 7-day-old (P7) WT and KO mice demonstrate identical baseline levels of both P-Akt and P-ERK. **(B)** The levels of P-Akt protein induced by a 30-min pulse of IGF1 was quantified in WT and KO GNPs by Western blot with densitometric analysis, and no significant differences were found between genotypes (*C* = control; *I* = IGF1). **(C)** IGF1 induced robust, but similar increases in P-Akt in both WT and KO GNPs after a 15-min pulse. This effect was attenuated at 4 h and 8 h, and no activity was found at 24 h (data not shown). P-ERK, on the other hand, appeared constitutively activated in these culture conditions, with no change in levels in either genotype following IGF1 treatment. **(D)** Phospho-S6 kinase, which is downstream of Akt, was markedly upregulated in KO GNPs pulsed 30 min with IGF1 compared to untreated GNPs. In contrast, IGF1 failed to upregulate phospho-S6 kinase in WT GNPs (though it robustly increased P-Akt), suggesting *En2* may be an important negative regulator of the S6 kinase pathway. Total S6 kinase protein levels were not different between genotypes (not shown). Densitometry quantification in **B** and **D** is expressed in arbitrary units as percent control ± SEM. *n* = 3 experiments per genotype (3 animals per experiment, per genotype); #, Significance compared to genotype control, *p* ≤ 0.05; **, significance compared across genotypes, *p* ≤ 0.01.

As shown above, IGF1 is a pleiotropic signal in the developing cerebellum, regulating proliferation, neurite outgrowth and survival via its tyrosine-kinase receptor, the IGF1 receptor (IGF1R), which is activated through receptor autophosphorylation of tyrosine residues [60]. Activated IGF1R in turn binds and activates one of several phosphorylation cascades engaging different signal transduction pathways, with phosphotidylinositol-3-kinase (PI3K) being one of the most commonly activated in cerebellar GNPs [61-63]. PI3K can induce activation of phosphokinase B/Akt (Akt), leading to inhibition of glycogen synthase-kinase-3-beta (GSK3beta), and can promote proliferation and survival [64,65]. Alternatively, IGF1 signaling can activate ras/raf-MEK, which activates the mitogen-activated protein kinase (MAPK)/extracellular signal-regulated protein kinase 1 and 2 (ERK1/2), leading to survival and differentiation [66]. To determine which cascades may underlie genotype-specific effects of IGF1, protein levels and the activation ratios of phospho-Akt/Akt (P-Akt) and phopho-ERK1/2/ERK1/2 (P-ERK) were measured by Western blot. Initial comparisons were made between untreated, whole cerebellar lysates from P7 KO and WT mouse pups. There were no genotype differences in either P-Akt or P-ERK, suggesting that in the absence of *En2,* baseline pathway activation was similar to WT in developing cerebellum (Figure 4A).

To determine whether IGF1 signaling through PI3K-Akt or MAPK is altered specifically in GNPs in the absence of *En2*, cells were isolated from each genotype and cultured at cell densities similar to those used in the ^3^H-dT proliferation assays. To assess IGF1 activity, KO and WT GNPs were cultured in defined media for 2 h without IGF1 and were then pulsed for 30 min with vehicle or 10 ng/ml IGF1. Both KO and WT GNPs exhibited almost no P-Akt activation in vehicle-treated control cultures (Figure 4B). Conversely, IGF1 elicited identical, robust phosphorylation of Akt in both genotypes. Previous studies show that PI3K pathway activation, measured by phosphorylation of Akt, occurs within minutes of growth factor treatment and becomes maximal at 1 h [67]. However, our culture results on mitosis and differentiation were obtained following continuous growth factor exposure for 24 h. Therefore, experiments were repeated at several later time points, including IGF1 treatments of 15 min, 4 h and 8 h. Though IGF1 increased P-Akt early at 15 min, and to a lesser degree at 4 h and 8 h, there were no differences between the genotypes; further, phospho-GSK3-beta protein levels were also no different between genotypes (data not shown). Furthermore, IGF1 pulses did not significantly increase P-ERK at any time point, nor were P-ERK levels different between the genotypes (Figure 4C). This suggests that in our culture models, IGF1 preferentially activates the PI3K-Akt pathway, whereas the MAPK pathway is constitutively activated, similar to previous reports [68]. Genotype-specific differences in IGF1 responses do not appear to be mediated by these dominant upstream pathways.

Given the dominance of the PI3K pathway in our culture models, differences in *En2* KO and WT GNP responses to IGF1 were explored further downstream of Akt. The downstream Akt target, mammalian target of rapamycin (mTOR), is known to regulate cell cycle progression as well as protein translation via activation of S6 kinase (S6K) and eukaryotic initiation factor 4B (eIF4B) [69]. In contrast to the upstream signals, IGF1 induced a differential genotype response in phospho-S6 kinase (P-S6K), eliciting a 25-fold increase in P-S6K levels in KO GNPs at 30 min, but inducing no significant increase in WT GNPs (Figure 4D). These data suggest that the differential genotype response of WT and KO GNPs to IGF1 may reflect a previously undefined interaction between *En2* and downstream effector molecules of the IGF1-PI3K-Akt-mTOR signaling cascade.

### Overexpression of *En2* cDNA promotes cell cycle exit and differentiation

Given our findings that the absence of *En2* resulted in increased GNP proliferation and decreased differentiation, it follows that overexpression should elicit opposite effects: increased GNP cell cycle exit and differentiation. Previously, we demonstrated that ectopic *En2* overexpression in embryonic cortical precursors altered neurogenesis [7], and additional studies in HEK293 cells confirmed that transfection produces overexpression of *En2* cDNA (not shown). Using these same vectors, we overexpressed *En2* by lipid transfection of P7 GNPs.

**Figure 5:**
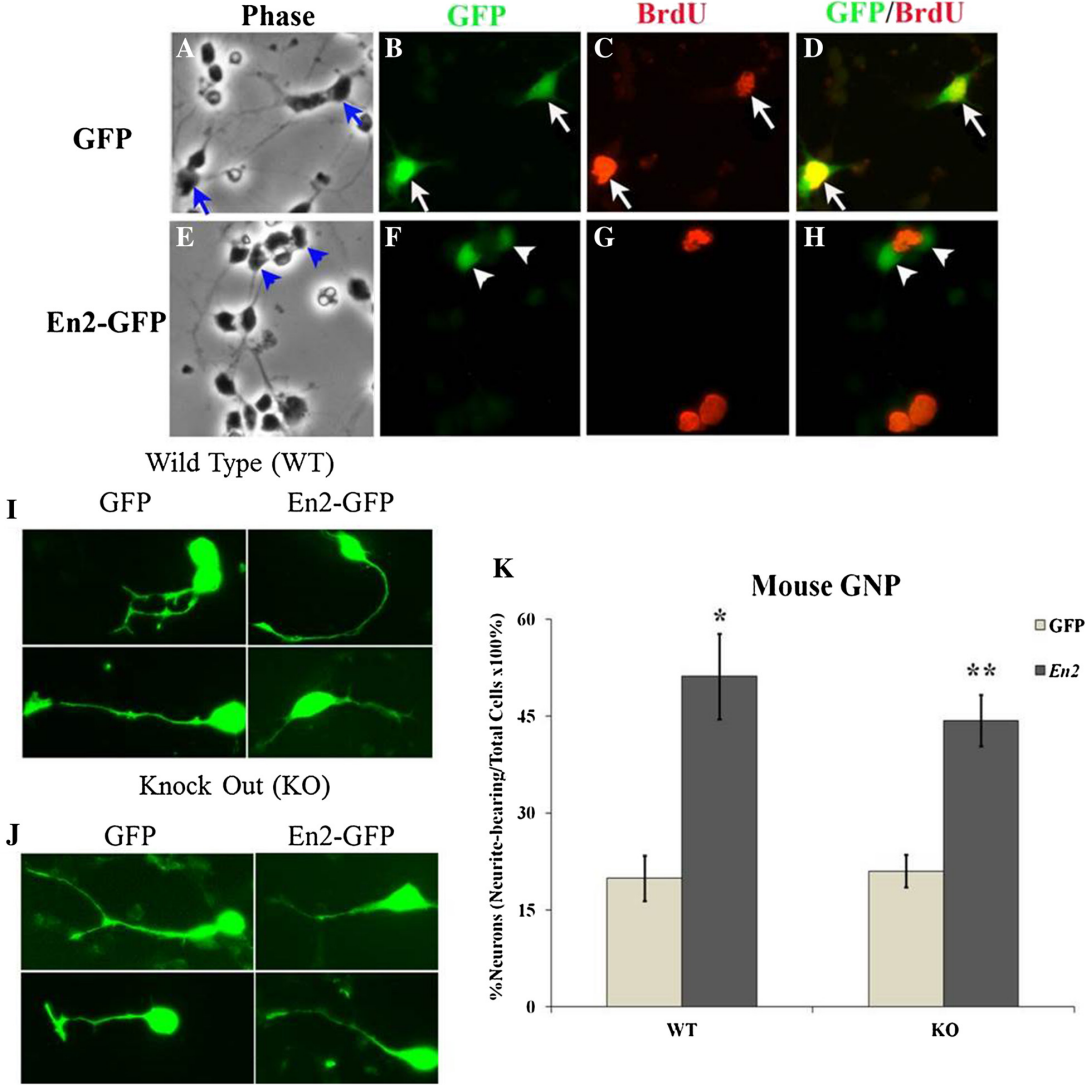
**Overexpression of *En2 *cDNA decreases mitotic labeling and increases neurite outgrowth of P7 mouse GNPs.** P7 mouse GNPs were transfected for 5 h with GFP control or *En2*-GFP cDNA vectors and 24 h later following BrdU treatment were fixed and immunostained for BrdU. Transfection efficiency was approximately 10% for each vector, with similar total numbers of transfected cells. Cells are shown (400×) under phase **(A, E)** or fluorescence **(B-D; F-H)** microscopy, after transfection with GFP control vector **(A-D)** and *En2*-GFP vector **(E-H)**, revealing GFP (*green*; **B**, **F**), BrdU (*red*; **C**, **G**) and double labeling **(D, H)**. *Arrows* point to double-labeled cells in **A**-**D**, whereas *arrowheads* in **E**-**H** identify cells that express GFP only, separate from cells with BrdU signal. **(I)** Examples of GNPs from WT mice transfected with GFP control and *En2*-GFP vectors that extend neurites >2 cell somas exhibiting a range in size. **(J)** Examples of GNPs from KO mice transfected with GFP and *En2*-GFP vectors that extend neurites >2 cell somas. **(K)***En2* overexpression increased the proportion of GNPs exhibiting neuronal morphologies by 2.5-fold in WT cells as well as by two-fold in KO cells. *n* = 6-15, each vector, 3–5 experiments; *, significance between GFP and *En2*, *p* ≤ 0.05; **, *p* ≤ 0.01.

GNPs were isolated from P7 WT mice and transfected 2 h after plating with the *En2*-GFP cDNA vector or control GFP vector. Proliferation was assessed 24 h after the 5-h transfection period by a terminal 4-h pulse of BrdU, approximately 30 h post plating. While GNPs transfected with control GFP vector exhibited a BrdU LI of 6.54%, overexpression of *En2* completely abolished BrdU labeling (Figure 5A-H; *p* ≤ 0.05; *n* = 8 per vector). In addition to cell cycle withdrawal, *En2* overexpression elicited a 2.5-fold increase in WT GNPs exhibiting neuronal morphologies (i.e., neurite-bearing cells, Figure 5I, K) compared to GFP-transfected controls, indicating that *En2* overexpression promotes cell cycle exit and early differentiation. Moreover, in GNPs from the KO mouse, *En2* overexpression elicited effects that were nearly identical to WT cells, demonstrating a two-fold increase in cells exhibiting neuronal morphologies (Figure 5J,K). These data suggest the deficits in neurite outgrowth observed in KO GNPs (Figure 3) that are due to the developmental absence of *En2* were rescued by acute re-expression of the transcription factor at P7. Thus, as predicted, *En2* overexpression produced effects opposite to *En2* deletion, reducing markers of proliferation while increasing markers of differentiation.

**Figure 6:**
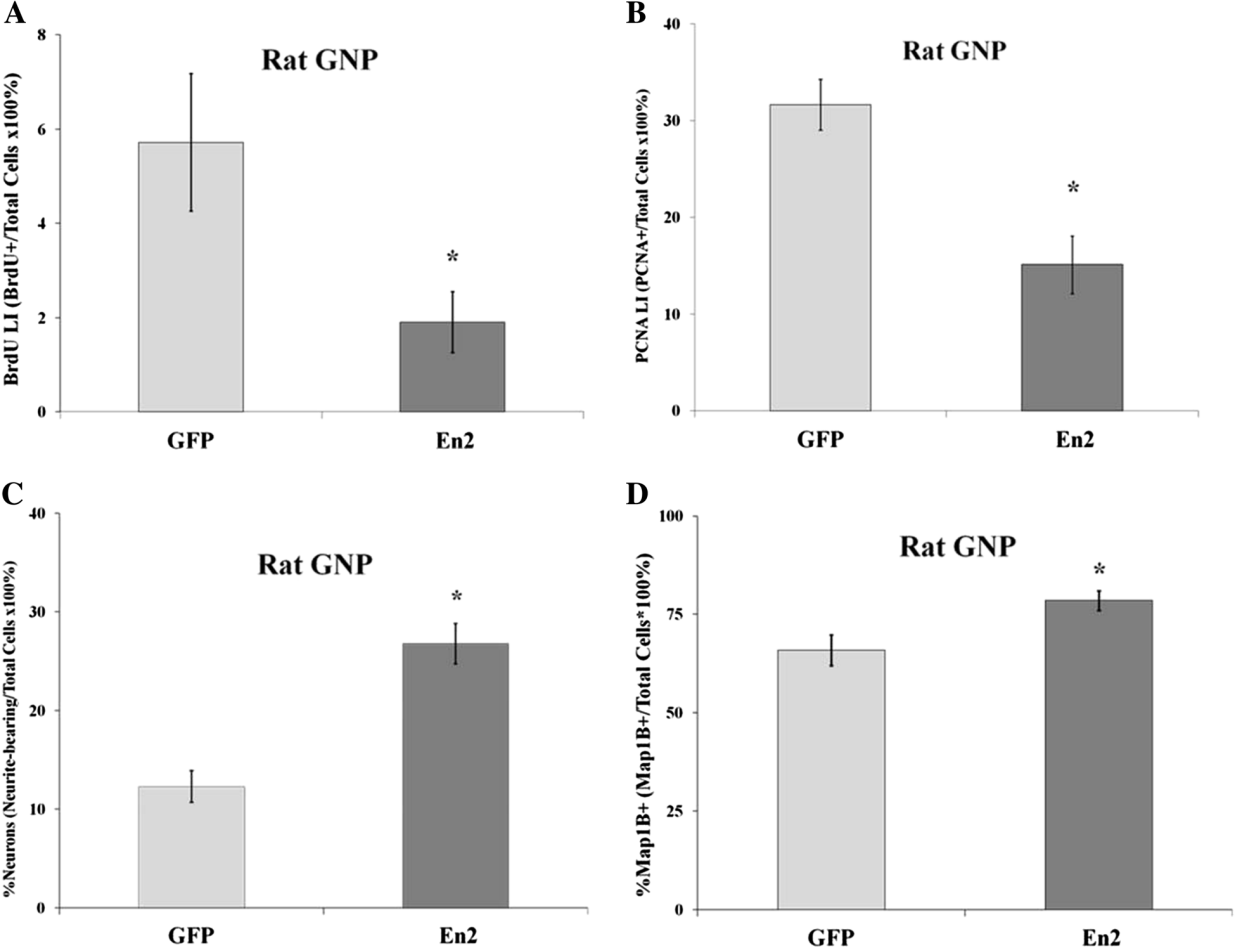
***En2 *cDNA overexpression in P7 rat GNPs also elicits reduced proliferation and increased neurite outgrowth. (A)** Following *En2* overexpression, the BrdU LI of rat GNP at ~30 h was reduced by 67%. **(B)** PCNA expression was reduced 52% in P7 rat GNPs following *En2* overexpression; *n* = 6 each vector. Data are expressed as percent PCNA + GFP+/total GFP + cells. *N* = 6-12 for each vector, 3 experiments. *,*p* ≤ 0.05. **(C)** Overexpression of *En2* in rat GNPs doubled the proportion of cells with neuronal morphologies, replicating results observed in mouse. **(D)** Overexpression of *En2* in rat GNPs increased the proportion of Map1b + cells among all GFP + cells, including neurite-bearing and non-neurite-bearing cells, suggesting *En2* may serve to promote differentiation prior to the onset of neurite outgrowth. *n* = 6-15, each vector, 3–5 experiments; *, significance between GFP and *En2*, *p* ≤ 0.05.

To further explore *En2* effects, we performed additional studies in postnatal rats, which exhibit very similar cerebellar development [70,71], because several antibodies were ineffective for murine cells, and postnatal rats were more readily available. *En2* DNA and protein sequences are highly homologous across mammalian species, with ~96% homology between mouse and rat [72]. As observed in mouse, rat GNPs overexpressing *En2* also exhibited a reduction in BrdU LI, by 67%, compared to GFP-only transfected cells (Figure 6A). However, changes in BrdU labeling may possibly reflect changes in cell cycle stage lengths. To more directly assess precursor status, we immunostained GNPs for proliferating cell nuclear antigen (PCNA). In contrast to BrdU, PCNA is expressed from S- through M-phase and has a long half-life, making it a more sensitive marker of the precursor cell compartment [73]. More than 30% of GNP exhibited PCNA immunoreactivity (Figure 6B), suggesting that many cells were proliferative precursors. Overexpression of *En2* reduced PCNA immunostaining by 52% (Figure 6B), suggesting that gene overexpression induced cells to transition from precursors to postmitotic neurons. Additionally, similar to mouse GNPs, overexpression of *En2* in rat GNPs resulted in a two-fold increase in cells exhibiting neuronal morphologies (Figure 6C). Furthermore, neurite-bearing cells characteristically express cytoskeletal proteins such as microtubule-associated protein 1b (Map1b), which is a known target for regulation by *En*[74]. *En2* overexpression increased Map1b immunostaining in transfected GNPs by 27% (Figure 6D). Together, these data suggest *En2* overexpression promotes rat GNP cell cycle exit similar to mice, suggesting *En2* may have an evolutionarily conserved function in other mammalian species.

## Discussion

Based on both loss- and gain-of-function approaches, our studies suggest *En2* promotes GNP cell cycle exit and differentiation during postnatal cerebellar development. In the absence of *En2*, GNPs displayed increased proliferation markers *in vivo* and in culture. Further, GNPs elaborated fewer neuronal processes in response to growth factors but exhibited no differences in survival. Conversely, overexpression of *En2* promoted cell cycle exit and stimulated neurite outgrowth, an effect demonstrated in both mouse and rat GNPs. Finally, we identified previously unknown interactions of *En2* with IGF1 signaling. In the absence of *En2*, IGF1 elicited greater stimulation of DNA synthesis in culture and *in vivo*, effects associated with marked activation of phospho-S6K, while there were no changes in upstream PI3K and ERK signaling. Significantly, regulation of proliferation by other mitogenic signals was unaffected. These studies characterize *En2*’s roles in postnatal cerebellar GNP neurogenesis and differentiation and interactions with IGF1, which invite further study with respect to the role of *En2* in the pathogenesis of development diseases where cerebellar structures are affected, such as ASD and schizophrenia.

### *En2* promotes cell cycle exit

During postnatal cerebellar development, outer EGL GNPs proliferate robustly, producing a large population of internal granule layer neurons. Underlying Purkinje neurons regulate this process by producing extracellular growth factors, including Shh, IGF1 and PACAP [49,50,75]. However, cell intrinsic mechanisms also regulate the GNP transition from proliferation to post-mitotic differentiation [76]. While mechanisms remain undefined, we present evidence suggesting *En2* serves to promote GNP cell cycle exit. These findings parallel evidence that outer EGL GNPs that are devoid of *En2* remain proliferative while inner EGL GNPs begin expressing *En2* as they exit the cycle [15,24]. Indeed, when isolated in culture, GNPs from the WT mouse were less proliferative than those from the KO. These observations suggest *En2* normally inhibits cell cycle progression in a cell-autonomous fashion, a model that is also supported by *En2* overexpression that reduces S phase entry and precursor PCNA expression.

### *En2* modulates IGF1 signaling

Purkinje neurons secrete growth factors, such as Shh, FGF, IGF1, BDNF and PACAP, that support local GNP proliferation, survival and differentiation [37,46,49,52,65,77-79]. We find *En2* expression modulates GNP responses to IGF1, suggesting that the onset of *En2* expression contributes to cell cycle exit and differentiation in the growth factor-rich environment of the EGL. In *En2* KO GNPs, IGF1 induced two- to three-fold greater increases in DNA synthesis in culture and three-fold greater stimulation of cerebellar DNA synthesis *in vivo*. Importantly, *En2* did not appear to regulate overall cell cycle machinery or signaling by other growth factors that employ signaling systems similar to IGF1, such as tyrosine-kinase engaging receptors or PI3K/Akt or MAPK pathways. Rather, in the absence of *En2*, IGF1 markedly upregulated P-S6K, an effect not observed in WT cells. While P-S6K is a well-defined regulator of mitogen-induced cell cycle progression [69], there were no genotype differences in upstream PI3K-Akt-GSK3beta or MEK/ERK pathways. To our knowledge, this is the first report of interactions between *En2* expression and IGF1 signaling.

**Figure 7:**
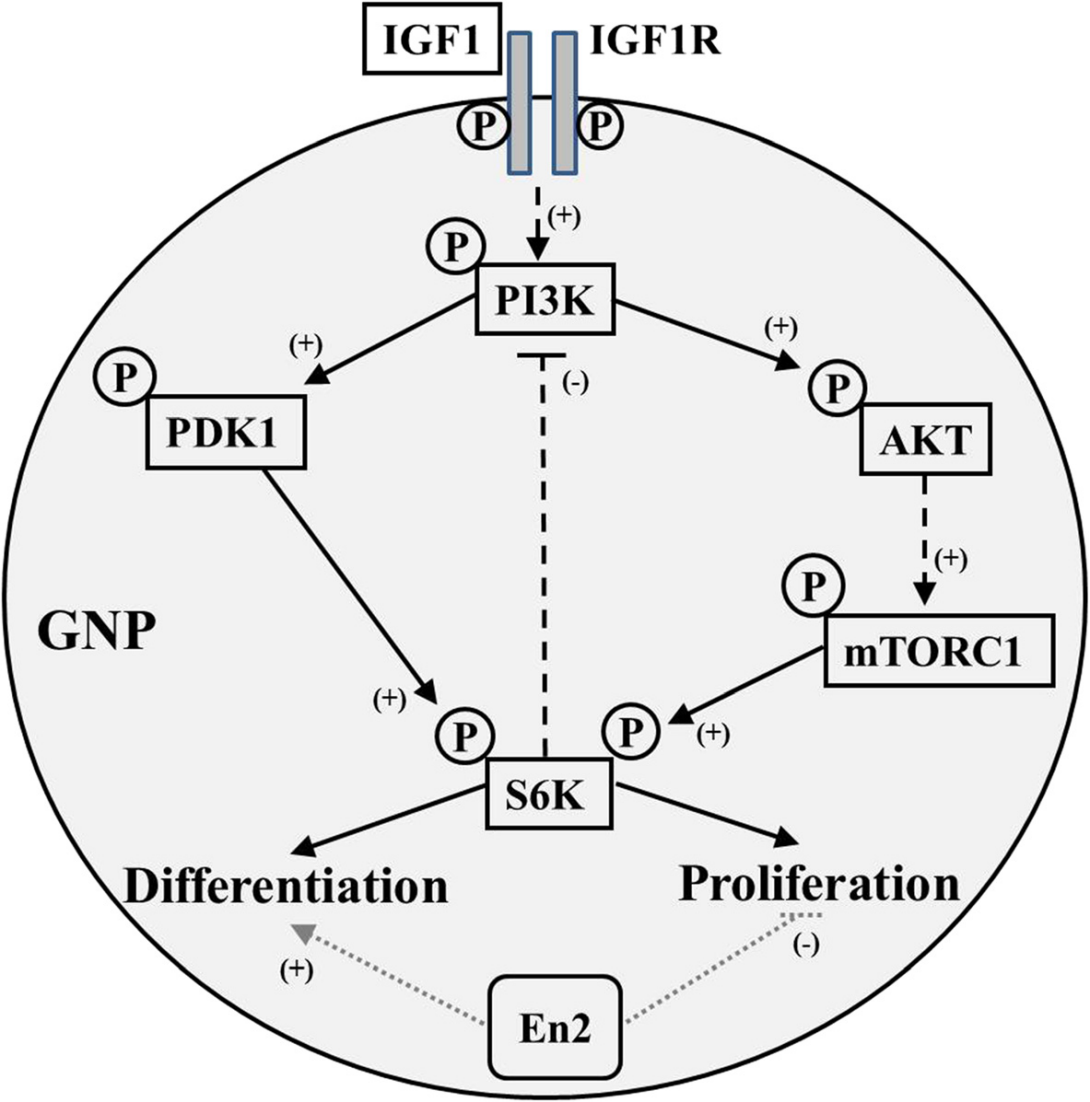
***En2 *modulates IGF1’s pleiotropic effects in GNPs, potentially through altered S6 kinase activation.** While *En2* expression appeared to have no effect on IGF1 activation of the PI3K pathway at the level of Akt phosphorylation, the absence of *En2* caused increased phosphorylation of S6 kinase. Thus, we propose a model in which *En2* expression in postnatal GNPs promotes differentiation and inhibits proliferation via disruption of S6 kinase activation, a well-characterized promitogenic signal. Potential targets for *En2* may include PDK1 and mTORC1, which directly phosphorylate S6K, though these interactions remain to be explored. Alternatively, *En2* may alter feedback inhibition between the S6K and the PI3K pathway. *Dashed lines* signify indirect pathways. *Arrows* and (+) indicate activation. *Flat head* and (-) indicate inhibition. *GNP* = Granule neuron precursor. *IGF1R* = Insulin-like growth factor-1 receptor. *P* = Phosphorylation.

These initial observations require further study to (1) define relationships of P-S6K activation to enhanced mitogenesis and (2) identify mediating upstream regulators, potentially members of the mTOR complex 1 family [69,80,81]. Indeed, extracellular mitogens promote mTOR signaling, which acts through S6K and eIF4B to promote cell proliferation [80]. eIF4B phosphorylation by S6K in turn is growth-factor dependent and important for promoting ribosome-mRNA binding, and consequent growth and proliferation [69]. Significantly, Engrailed 2 protein has previously been shown to bind eIF4E [82]. Thus, Engrailed 2 may interact with other members of the eIF4 family, such as eIF4B, providing a mechanism through which *En2* alters downstream mTOR signaling. This might explain the increases in IGF1-induced proliferation we observe both *in vitro* and *in viv*o. However, what underlies the increased IGF1-induced S6K phosphorylation in *En2* KO cells? Independent of Akt-mTOR signaling, IGF1 activates through PI3K the 3-phosphoinositide-dependent protein kinase 1 (PDK1), which is known to phosphorylate S6K (Figure 7) [83]. Thus, PDK1 is a candidate in addition to mTOR for further study. Furthermore, since S6K itself negatively regulates IGF1 signaling by phosphorylating the insulin response substrate 1 (IRS1) [84], *En2* may disrupt this inhibitory feedback loop, leading to increased S6K phosphorylation and activation via mTOR-dependent and -independent pathways downstream of PI3K. Thus, our data suggest *En2* may function normally to attenuate mTOR-mediated proliferation in a growth factor-rich environment, though the mechanisms by which this may occur remain to be explored.

In addition to altered mitogenesis, *En2* KO GNPs grew fewer neurites in response to neuritogenic factors, IGF1 and PACAP, though their combination synergistically overcame differentiation deficits. Decreased IGF1-induced neuritogenesis was not merely due to failed cell cycle exit, a possibility because the PACAP rescue of reduced neurite outgrowth was accompanied by inhibition of proliferation [38]. Rather, treatment with another anti-mitogen, FGF2, did not rescue the differentiation defect, but in fact attenuated IGF1-stimulated neuritogenesis in both genotypes. Significantly, GNP survival was also not compromised in the absence of *En2*, nor was growth factor-induced trophism. In aggregate, these data suggest that a complex array of mitogenic, trophic and differentiative signals within the EGL act in concert with cell-autonomous gene expression (i.e., *En2* expression) to execute GNP maturation.

Previous evidence indicates that multiple interactions of intrinsic and extrinsic signals control GNP proliferation. With regard to extrinsic signals, we previously found that PACAP inhibits Shh-induced GNP mitosis by upregulating adenylate cyclase [38], whereas others show similar roles for bone morphogenetic proteins via Smad [53,85]. Conversely, in the current study, we find IGF1 and Shh synergistically stimulate DNA synthesis. Furthermore, interactions between intrinsic signals and growth factors are also known. For example, transcription factor Atoh1/MATH1 expressed in EGL precursors promotes Shh-induced proliferation [86] by regulating expression of downstream target, Gli1 [87]. Similar promitogenic effects have been defined for Zic family members, including 1, 2 and 4, as well as ATF5 [88-90]. On the other hand, ZNF238/RP58 is expressed as GNPs exit the cell cycle, and overexpression inhibits proliferation [91,92], findings that parallel our observations with *En2*. Thus, a number of gene-growth factor interactions likely contribute to GNP development. That no other mitogenic or anti-mitogenic signal elicited genotypic differences in *En2* KO and WT GNPs suggests there is a specific yet indirect interaction between *En2* and IGF1 signaling (Figure 7), with one feature being altered S6K activation. The expression of *En2* by GNPs may facilitate their transition from proliferation to differentiation in an environment rich in mitogenic signals [93], thereby allowing subpopulations of cells to continue cycling while others exit and differentiate. *En2* expression has been reported to be greater in the vermis than the hemispheres [15,24], which may be one mechanism explaining the delay between vermis and hemisphere GNP proliferation during development [23].

### *En2* overexpression promotes cell cycle exit and differentiation

Previously, we found that ectopic *En2* overexpression in embryonic cortical precursors increased proliferation [7]. This stimulatory effect, opposite to what we observe here, is consistent with the universal expression of *En2* in prenatal hindbrain progenitors and suggests that before birth *En2* serves to maintain proliferation. In the current study of postnatal GNPs, *En2* overexpression completely abolished BrdU labeling in WT mouse cells and reduced markers of proliferation (PCNA and BrdU) in rat GNPs. Additionally, *En2* overexpression more than doubled the proportion of mouse and rat GNPs that exhibited neurite outgrowth. Significantly, neurite outgrowth was stimulated even in KO GNPs, suggesting that acute *En2* expression may reverse morphological deficits associated with the absence of *En2* for all of development, though additional studies are warranted. Furthermore, despite biological differences in GNPs from mice and rats [38], *En2* overexpression produced similar phenotypes in both species, suggesting *En2* function is evolutionarily conserved. The opposite consequences of *En2* deletion and overexpression on GNP maturation suggest *En2* promotes cell cycle exit and differentiation during postnatal cerebellar development.

One limitation to our current model, however, remains the unexplored role of *En2* in prenatal cerebellar neurogenesis. While postnatal granule neurogenesis ultimately dictates final cerebellar morphology and size, prenatal patterning events organize the developing cerebellum and delineate neuronal progenitor cell populations, ultimately producing a cytoarchitectural framework for future axonal pathway elaboration [15,94-97]. In the early cerebellar anlagen, *En2* expression is ubiquitous, as is progenitor cell proliferation; therefore, *En2* is unlikely to promote cell cycle exit and differentiation at this time. Previous studies suggest *En2* may function during this period to specify numbers as well as types of progenitor cells that will give rise to various cerebellar neuronal populations [14,16,50,95,96,98]. Thus, our characterization of postnatal *En2* function is likely a time-locked developmental phenomenon that cannot address its prenatal activities. Rather, our demonstration of increased GNP proliferation may potentially suggest why *En2* KO mice are capable of producing cerebella at all, despite the prenatal insult.

### *EN2* and ASD

We reported previously that intronic polymorphisms (A-C haplotype) of human *EN2* are associated with ASD [7,9] and that the presence of these SNPs results in altered transcription factor binding as well as increased levels of gene expression [99]. Furthermore, our recent studies indicate that in human cerebellum, the disease-associated allele produces increased *EN2* expression [100]. If the *En2* activity defined here in rodent studies is relevant to primates, how then would altered *EN2* expression be expected to affect humans? Increased *EN2* expression during postnatal cerebellar development, the period when the majority of human granule neurons are generated, would likely produce a granule neuron deficit by eliciting premature cell cycle exit. However, as with the mice, such a simple prediction is unwarranted because the prenatal effects of gene alteration remain undefined. But at the broader level, children with ASD exhibit a range of cerebellar structural abnormalities, including a diminished vermis as well as enlarged hemispheres in which granule neuron numbers are dysregulated [3,101-103], a phenotype to which *EN2* may contribute. Indeed, in mouse models, both gene deletion as well as ectopic overexpression produces cerebellar hypoplasia [13,18,20]. In addition, cerebellar abnormalities have been described in human and mouse studies of autistic phenotypes in tuberous sclerosis as well as a number of neuropsychiatric disorders including schizophrenia, attention deficit hyperactivity disorder, and cognitive and language disabilities [35,104-106].

Another interesting implication of our studies is identification of an interaction between *En2* expression and IGF1, the latter being an important pleiotropic growth factor associated with disease as well as a possible therapeutic target. Riikonen *et al*. [107] reported reduced IGF1 in cerebrospinal fluid (CSF) of ASD children compared to age-matched controls and that CSF levels correlated with head circumference in ASD, but not control children. Given the *En2*-IGF1 interaction we describe, one might speculate whether altered *EN2* expression may coincide with abnormal IGF1 to contribute to ASD pathogenesis, a question that remains to be investigated. From a therapeutic perspective, the Akt-mTOR-S6K pathway is dysregulated in multiple animal models of monogenic causes of ASD including fragile X mental retardation [108], Rett syndrome [109] and tuberous sclerosis [110], whereas IGF1 ligands may improve neurodevelopmental symptoms in Rett [111] and the SHANK3 autism-related mouse model of Phelan-McDermid syndrome [112]. It is intriguing that yet another autism-associated gene, in this case *EN2*, implicates disordered Akt-mTOR-S6K signaling in the disease phenotype [113]. The exact molecular mechanisms mediating *En2*’s modulation of IGF1 signaling remain to be elucidated.

## Conclusions

Our current studies aimed to define the function of one autism-associated gene, *EN2*, at a specific time during postnatal brain development. Using both a loss of function KO mouse and a gain of function cDNA overexpression vector, we demonstrated that *En2* promotes postnatal cerebellar GNP cell cycle exit and differentiation. Further, we characterized a previously unknown interaction between *En2* and an important developmental growth factor, IGF1, and demonstrated downstream signaling pathway activation through S6K, a target of the mTOR pathway. These data add to the understanding of postnatal cerebellar development and the complex gene-growth factor interactions that regulate cell biologic processes such as proliferation and differentiation. Further, they provide insight into a possible pathogenetic mechanism by which ASD-associated alleles in human *EN2* may alter neurodevelopment during a critical period in susceptible patients. While further investigation remains to define the mediators of the phenomena described here, these observations add further support to a previously recognized signaling pathway as a potential target for therapy.

## Abbreviations

ASD: Autism spectrum disorder; BDNF: Brain-derived neurotrophic factor; BrdU: Bromodeoxyuridine; CSF: Cerebrospinal fluid; DNA: Deoxyribonucleic acid; EGF: Epidermal growth factor; EGL: External germinal layer; En2: *Engrailed 2*; ERK1/2: Extracellular signal-regulated protein kinase 1/2; FGF: Fibroblast growth factor; eIF4B/eIF4E: Eukaryotic initiating factor 4B/E; GNP: Granule neuron precursor; GSK3beta: Glycogen synthase kinase 3-beta; IGF1: Insulin-like growth factor 1; IRS1: Insulin response structure 1; KO: Knock out; MAP1b: Microtubule-associated protein 1b; MAPK: Mitogen activated protein kinase; MRI: Magnetic resonance imaging; mTOR: Mammalian target of rapamycin; PACAP: Pituitary adenylate cyclase activating peptide; PCNA: Proliferating cell nuclear antigen; PDK1: 3-phosphoinositide-dependent protein kinase 1; PI3K: Phosphotydilinositol-3-kinase; Shh: Sonic hedgehog; SNP: Single nucleotide polymorphism; VEGF: Vascular endothelial growth factor; WT: Wildtype.

## Competing interests

The authors declare that they have no competing interests.

## Authors’ contributions

IR conducted the majority of experiments including cloning of murine *En2*, creating the transfection vector, dissections and cultures, transfections, immunohistochemistry, western blots and data analysis, and wrote and edited the manuscript for submission. LL conducted experiments characterizing phospho-S6K activation in KO and WT mice. KM performed immunocytochemistry and data analysis for transfection experiments. MDG prepared cerebellar sections and performed immunohistochemistry and analysis of in vivo BrdU experiments. EVB conducted and performed immunocytochemical analyses of initial transfection experiments in both cortical and cerebellar cultures. SK propagated, maintained, time-mated, and genotyped *En2* mutant and WT mouse colonies. JM oversaw genetic analyses of mice, cloning and production of transfection vectors, provided intellectual guidance of experimental design, and participated in the writing and editing of the final manuscript for submission. EDB conceived of the original experimental model, provided intellectual and financial support for the development and execution of all experiments, and participated in the writing and editing of the manuscript for final submission. All authors read and approved the final manuscript.
